# The complex association between the antioxidant defense system and clinical status in early psychosis

**DOI:** 10.1371/journal.pone.0194685

**Published:** 2018-04-26

**Authors:** Saínza García, Susana Alberich, Mónica Martínez-Cengotitabengoa, Celso Arango, Josefina Castro-Fornieles, Mara Parellada, Inmaculada Baeza, Carmen Moreno, Juan Antonio Micó, Esther Berrocoso, Montserrat Graell, Soraya Otero, Tatiana Simal, Ana González-Pinto

**Affiliations:** 1 Centre for Biomedical Research in the Mental Health Network (CIBERSAM), Madrid, Spain; 2 BioAraba Research Institute, OSI Araba, Department of Psychiatry, Araba University Hospital, Vitoria, Spain; 3 University of the Basque Country, Vitoria, Spain; 4 Psychobiology Department, National Distance Education University (UNED), Vitoria, Spain; 5 Child and Adolescent Psychiatry Department, Gregorio Marañón General University Hospital, IiSGM, Madrid, Spain; 6 School of Medicine, Complutense University, Madrid, Spain; 7 August Pi i Sunyer Biomedical Research Institute (IDIBAPS), Barcelona, Spain; 8 Department of Child and Adolescent Psychiatry and Psychology, SGR489, Institute of Neuroscience, Hospital Clínic of Barcelona, Barcelona, Spain; 9 Department of Psychiatry and Clinical Psychobiology, University of Barcelona, Barcelona, Spain; 10 Neuropsychopharmacology & Psychobiology Research Group, University of Cádiz, Cadiz, Spain; 11 Institute of Research and Innovation in Biomedical Sciences, INiBICA, Cádiz, Spain; 12 Child and Adolescent Psychiatry and Psychology Department, Niño Jesús University Children's Hospital, La Princesa Institute for Health Research, Madrid, Spain; 13 Department of Psychiatry, Marqués de Valdecilla University Hospital, IDIVAL, Santander, Spain; 14 Child and Adolescent Psychiatry Unit, Marqués de Valdecilla University Hospital, Santander, Spain; 15 School of Medicine, University of Cantabria, Santander, Spain; 16 Department of Psychiatry, Miguel Servet University Hospital, Zaragoza, Spain; Maastricht University, NETHERLANDS

## Abstract

Oxidative stress is a pathophysiological mechanism potentially involved in psychiatric disorders. The objective of this study was to assess the relationship between total antioxidant status (TAS) and the functional status of patients with a first episode of psychosis at the onset of the disease. For this purpose, a sample of 70 patients aged between 9 and 17 years with a first episode of psychosis were followed up for a period of two years. Blood samples were drawn to measure TAS levels at three time points: at baseline, at one year, and at two years. Clinical symptoms and functioning were also assessed at the same time points using various scales. Linear regression analysis was performed to investigate the relationship between TAS and clinical status at each assessment, adjusting for potential confounding factors. The distribution of clinical variables was grouped in different percentiles to assess the dose-response in the relation between clinical variables and TAS. At baseline, patient's score on Children's Global Assessment Scale (CGAS) was directly and significantly associated with TAS with a monotonic increase in percentiles, and surprising this association was reversed after one and two years of follow-up with a monotonic decrease. In summary at the onset of the illness, TAS is positively related to clinical status, whereas as the illness progresses this correlation is reversed and becomes negative. This may be the result of an adaptive response.

## Introduction

Oxidative stress is a pathophysiological mechanism potentially involved in schizophrenia [1–5]. There is evidence that patients who have experienced just a single episode of psychosis have increased levels of lipid peroxidation [6–9] and a decreased total antioxidant capacity [10].

The total antioxidant activity of extracellular fluid can be calculated by adding endogenous and food-derived antioxidants. Total antioxidant status (TAS) is considered to have great potential in the search for biomarkers of functional damage in psychiatric disorders, given its association with the pathophysiology of schizophrenia spectrum disorders [1–3].

A number of case-control studies have been conducted to assess TAS using a cross-sectional method [10–13]. However, very few studies have explored the relation between TAS and the functionality of patients over time, with inconsistent results [12,14–16]. In addition, there are no published data on TAS at the onset of psychotic illness.

The objective of our study was to assess the relationship between total antioxidant status (TAS) and the functional status of patients with a first episode of psychosis (FEP), at the early course of the disease. We hypothesized that antioxidant status would be associated with both, the short and long-term functioning and clinical outcome in these patients.

## Materials and methods

### Study population

The child and adolescent first-episode psychosis study (CAFEPS) is a cohort study that included 110 patients with FEP aged between nine and 17 years at first assessment. FEP was defined as the presence of positive psychotic symptoms of delusions or hallucinations for a period of less than six months. The exclusion criteria for the patients were: presence of a concomitant Axis I disorder at the time of evaluation that might account for the psychotic symptoms (such as substance abuse, autistic spectrum disorders, post-traumatic stress disorder, or acute stress disorder), mental retardation (MR) per the DSM-IV criteria, including not only an IQ below 70 but also impaired functioning, pervasive developmental disorder, neurological disorders, history of head trauma with loss of consciousness, and pregnancy. Occasional substance use was not an exclusion criterion if positive symptoms persisted for more than 2 weeks after a negative urine drug test. Information about the sample and protocol has been described in detail elsewhere [17]. The study was approved by the Clinical Research Ethics Committee of all participating hospitals: Ethics and Clinical Research Boards of Gregorio Marañón General University Hospital, Clinical Research Ethics Committee of the Hospital Clínic de Barcelona, Clinical Research Ethics Committee of Euskadi, Clinical Research Ethics Committee of University of Navarra Clinic, Bioethics Committee of the Marqués de Valdecilla University Hospital and the Clinical Research Ethics Committee of Niño Jesús University Children's Hospital. Parents or legal guardians gave written informed consent and patients consented to participate in the study.

### Study design and clinical assessments

We conducted a prospective two-year follow-up study to assess the TAS and clinical status of FEP patients at three time points (at baseline and at one and two years). Clinical assessment was performed by trained clinicians who used the following scales: 1) the Spanish version of the Positive and Negative Syndrome Scale (PANSS)[18], which measures the severity of these types of symptoms; 2) the Hamilton Rating Scale for Depression (HRSD) [19], which measures the severity of depressive symptoms; 3) the Young Mania Rating Scale (YMRS), which measures the severity of manic symptoms [20]; and 4) the Children's Global Assessment Scale (CGAS) [21], which rates a patient’s level of functioning and severity of symptoms on a scale of 0 to 100.

### Assessment of TAS

Total antioxidant capacity was assessed by measuring TAS in peripheral blood at the aforementioned three time points. Blood samples (10ml) were collected in heparin-containing tubes after enrolment between 8:00 and 10:00 _AM_ and were immediately processed as follows: tubes were centrifuged for 5 min at 400 g at 4°C. Plasma was collected and centrifuged for 15 min at 14,000 g at 4°C and then stored at -80°C until analysis. All samples were analyzed in a single batch. Baseline data has been published previously by Micó et al. [10]. For the present study, we only used baseline data from the subset of patients who were followed up. TAS was determined by standardized spectrophotometric assays (Bioxytech) in plasma. Briefly, the TAS assay relies on the ability of antioxidants present in plasma to inhibit the oxidation of ABTS (2,2’-azino-bis(3-ethylbenzthiazoline-6-sulphonicacid)), which is monitored by reading absorbance at 600 nm [22].

### Statistical analysis

After confirming the normality of sociodemographic and clinical data, a descriptive analysis was performed using means, standard deviation, and percentages. Analysis of variance (ANOVA) with repeated measures (measuring within subject variables) were used to analyse the main effect of time on TAS level, clinical parameters and antipsychotic dose. To investigate if some of the potential confounding factors (age, sex, ethnic group, diagnostic group, parental level of education, socioeconomic level, living arrangements, use of toxic substances and dose of medication in chlorpromazine equivalent unit) had influence on TAS levels in each visit, different statistical models were employed: T-student model for independent samples with categorical variables of two groups, analysis of variance (ANOVA) for categorical variables of more than two groups and bivariate Pearson correlation to compare continuous variables. To evaluate the relationship between TAS and clinical variables, linear regressions were performed and adjusted for the potentially confounding variables that revealed significant in the previous step. The final models contained (significant) confounding variables and the interaction of independent variables with these confounding variables was also included. To evaluate the relationship between TAS and scores on clinical scales, differential variables between baseline and 1-year/2-year values were calculated and linear regressions were performed. In addition, we used longitudinal linear models to analyse the relationship between the evolution of TAS and changes in clinical scale scores. These models were created in two steps: 1) we analysed the influence of the potential confounding variables on the evolution of TAS; 2) we defined the final models by including the variables that revealed significant in the first step and the clinical scales. Data are presented in terms of beta coefficients with *p* values and the corresponding 95% confidence intervals. The distribution of clinical variables was grouped in different percentiles to assess dose-response in the relation between clinical variables and TAS. All statistical analyses were carried out using SPSS v23.0 statistical software, with the significance level set at *p* <0.05.

## Results

### Characteristics of the sample

Of the 110 patients initially included in the study, 70 were selected to compose the final sample, as they had attended the baseline visit and at least the first follow-up visit. At the end of the study, there was a 35% reduction in the size of the sample with respect to the sample of the first follow-up year. The characteristics of the entire sample are detailed in Table 1. No significant changes were found in antipsychotic dose over time (F = 1.22; p>0.05). The scores obtained on the various scales are summarised in Table 2, which shows that scores had improved significantly on all scales at the end of the study (p<0.01).

**Table 1:** Sociodemographic characteristics and total antioxidant status (TAS).

*Sociodemographic characteristics*	*Patients (N = 70)*
Age in years, n (SD)	15.70 (1.63)
Sex (M/F), mean (%)	50 (71.4)/20(28.6)
Drug use, n baseline (%)	
Tobacco	20 (28.6)
Cannabis	20 (28.6)
Alcohol	17 (24.3)
Socioeconomic status, n (%)	
5 (lowest)	14 (20.0)
4	23 (32.9)
3	18 (25.7)
2	6 (8.6)
1 (highest)	9 (12.9)
Type of living arrangement, n (%)	
Birth parents	64 (91.4)
Alone	1 (1.4)
Other	5 (7.2)
Ethnic group, n (%)	
Caucasian	64 (91.4)
Hispanic	4 (5.7)
Other	2 (2.9)
Antipsychotic dose (chlorpromazine equivalent units), mean (SD)	
Baseline	260.66 (170.76)
1 year	267,99 (234,77)
2 years	213,42 (133,11)
*Variables related to oxidative stress*	
TAS (mM), mean (SD)	
Baseline	0.95 (0.30)
1 year	0.99 (0.42)
2 years	1.14 (0.36)

**Table 2:** Scores in the clinical assessments at the three time points.

	*Clinical assessment*		
	*Baseline*	*1 year*	*2 years*
PANSS Pos, mean (SD)**	24.2 (6.16)	12.63 (6.0)	12.11 (5.47)
PANSS Neg, mean (SD)**	21.07 (8.83)	16.54 (6.6)	14.69 (6.40)
PANSS Gen, mean (SD)**	47.01 (10.77)	29.63 (11.1)	27.46 (8.17)
PANSS Tot, mean (SD)**	92.3 (20.41)	58.80 (20.7)	54.27 (17.54)
YMRS, mean (SD)**	17.77 (11.46)	4.90 (7.6)	4.04 (5.20)
HDRS, mean (SD)**	19.31 (9.26)	5.76 (6.9)	4.88 (3.99)
CGAS, mean (SD)**	37.41 (14.22)	63.52(16.9)	67.11(18.13)

*p<0.05

**p<0.01

Pos (positive), Neg (negative), Gen (General), Tot (total)

### Relationship between oxidative stress and clinical assessment

#### Comparison of TAS among assessments

TAS improved progressively over the two years of follow-up, although none of the differences observed among the values measured at the three time points reached significance (Table 1). None of the potential confounding factors had a significant influence on TAS levels (S1 Table).

#### Relationship between TAS and clinical variables

**Fig 1:**
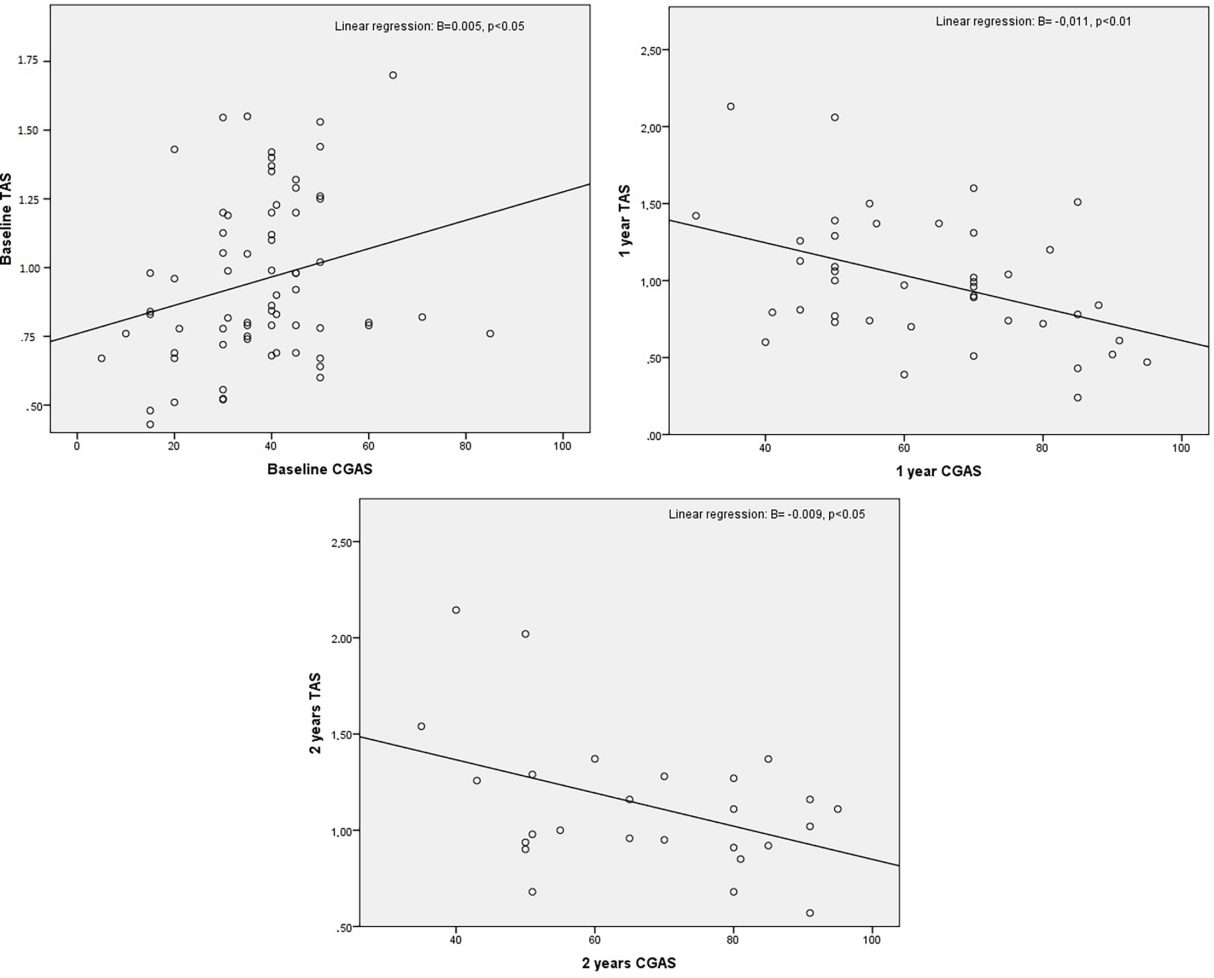
Relationship between total antioxidant status (TAS) and Children's Global Assessment Scale (CGAS) scores over a two year follow-up.

None of the variables included in the linear regression model as potential confounding factors were significant; hence, only clinical variables were included in the final model. At baseline, there was a significant positive relationship between TAS and CGAS scores. In contrast, at one year, this association was reversed, and TAS and YMRS and positive PANSS scores were observed to be all positively related. At two-year follow-up, TAS was significantly negatively correlated to CGAS scores and positively associated with YMRS, positive PANSS, negative PANSS, general PANSS and total PANSS scores (Table 3; Fig 1; S1 and S2 Figs). No correlation based on differential variables was observed between changes in TAS and variations in clinical scale scores. However, longitudinal linear models confirmed the previous observation of a relationship between TAS and CGAS (β = -0.006, p = 0.004, 95% CI: (-0.010, -0.002)), PANSS pos (β = 0.013, p = 0.040, 95% CI: (0.001, 0.025)) and YMRS scales (β = 0.014, p = 0.007, 95% CI: (0.004, 0.025)) after adjusting for gender. When CGAS were distributed in percentiles to analyse dose-response in this relation, a monotonic increase of the TAS was observed, with higher CGAS at baseline. In contrast, at one and two year follow-up, this monotonic effect was also significant, with a negative relationship between TAS values and CGAS (Table 4).

**Table 3:** Coefficients of linear regression models between clinical assessment and TAS level at each visit point.

*Clinical assessment*	*TAS*		
	*Baseline*	*1 year*	*2 years*
PANSS Pos	—	B = 0.024; p<0.05	B = 0.030; p<0.05
PANSS Neg	—	—	B = 0.025; p<0.05
PANSS Gen	—	—	B = 0.021;p<0.05
PANSS Tot	—	—	B = 0.011; p<0.01
YMRS	—	B = 0.018; p<0.05	B = 0.027; p = 0.05
HDRS	—	—	—
CGAS	B = 0.005; p<0.05	B = -0.011; p<0.01	B = -0.009; p<0.05

Pos (positive), Neg (negative), Gen (General), Tot (total)

**Table 4:** Effect size of CGAS on TAS (and 95% CIs).

	*BASELINE*		*1 YEAR*		*2 YEAR*	
Percentiles	CGAS	TAS Effect Size (95% CI)	CGAS	TAS Effect Size (95% CI)	CGAS	TAS Effect Size (95% CI)
**P3**	15	0.08 (0.002, 0.148)	35	-0.39 (-0.626, -0.144)	35	-0.32 (-0.573, -0.068)
**P25**	30	0.15 (0.003, 0.297)	50	-0.55 (-0.894, -0.206)	51	-0.46 (-0.828, -0.092)
**P50**	40	0.20 (0.004, 0.396)	63	-0.69 (-1.124, -0.256)	70.5	-0.64 (-1.149, -0.131)
**P75**	45	0.23 (0.010, 0.450)	80	-0.88 (-1.431, -0.329)	85	-0.77 (-1.383, -0.157)
**P97**	60	0.33 (0.012, 0.648)	95	-1.05 (-1.704, -0.396)	95	-0.86 (-1.545, -0.175)

## Discussion

**Fig 2:**
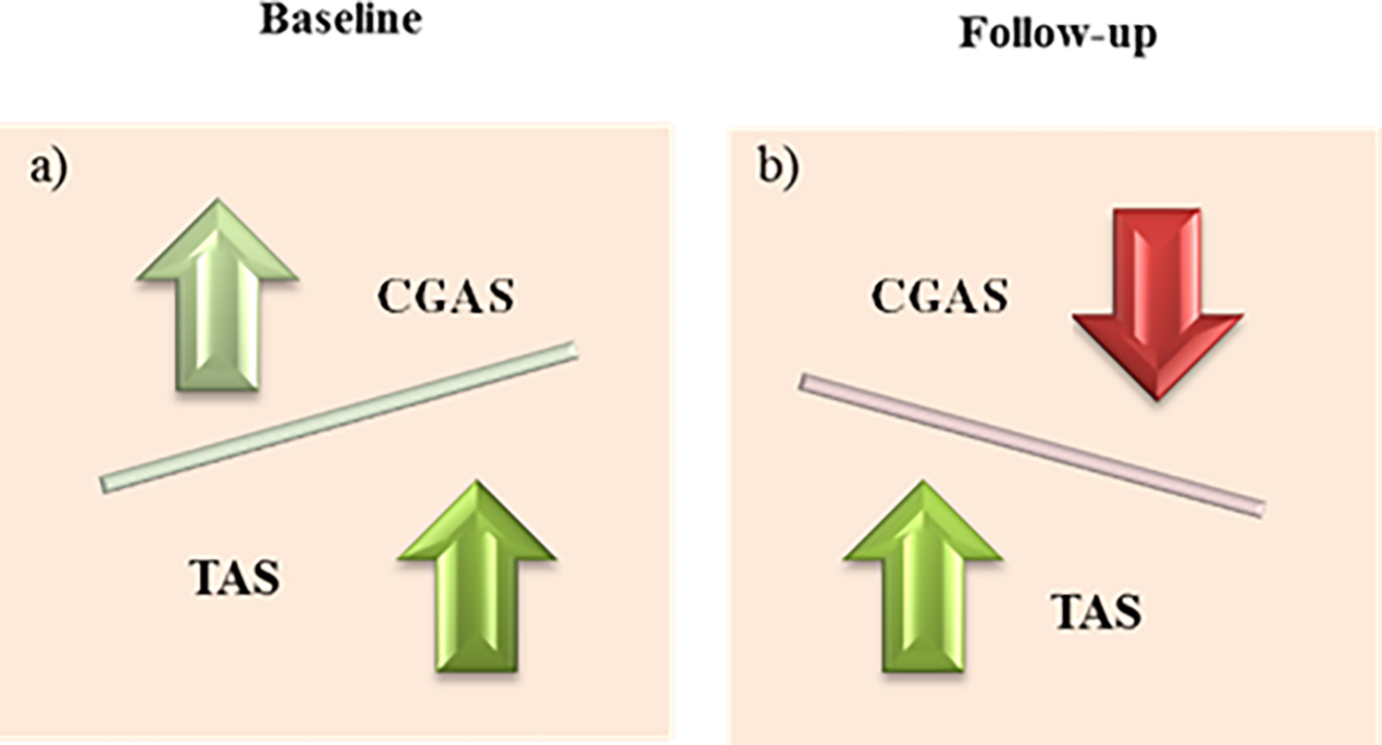
Relationship between CGAS score and TAS level at baseline and at two-year follow-up assessment. a) At baseline, there is a positive significant relationship between CGAS and TAS indicating that the better the patient's functioning, the higher his level of TAS; b) at one year and at two years of follow-up, this relationship is significantly reversed, with patients with the highest levels of TAS having the worst functionality.

To our knowledge, this is the first study to investigate the relationship between the antioxidant defence system and clinical and functional status in a sample of adolescents with FEP over time. Interestingly, the antioxidant reserve was smaller in the most severe cases at baseline, and higher in the most severe cases at follow-up, which may be the result of an adaptation of the antioxidant defence system in the long term. Specifically, we found that patients with the most severe positive psychotic symptoms and a worse functionality over two-year follow-up, exhibited higher levels of antioxidants as shown by the results obtained in the longitudinal linear models. These results are unlikely to be due to chance, as there is a monotonic increase in TAS levels at baseline with respect to functionality, and a monotonic decrease of TAS levels with respect to functionality over time (Fig 2).

We found no previous studies analysing these variables in children, and only Zhang et al. [12] have assessed the association between cognitive impairment and TAS in adult patients with schizophrenia. The authors observed that these variables were negatively correlated. In line with this, a previous study by Sánchez-Rodríguez et al. [15] comparing elderly people living in rural and urban areas revealed a significant negative relationship between cognitive performance and TAS, with those with the most severe cognitive impairment (lowest scores) having higher TAS.

The classical theory of hormesis suggests that short exposure to a low level of stress triggers protection mechanisms that help biological systems to recover baseline levels and cope better with high levels of stress [23]. In other words, it induces a homeostatic effect. This theory is also supported by some authors who have hypothesised that the greater the damage caused by oxidative stress in cells, the higher the antioxidant capacity of the organism [12,15,24,25]. At cellular level, these compensatory mechanisms occur through enzymatic regulation, which stimulates antioxidant response, thus increasing defence capacity mediated by the nuclear factor (erythroid-derived 2)-like 2 (Nrf2) [23,26,27]. Some studies have suggested that the activation of Nrf2 may reduce the susceptibility of neurons, astrocytes, oligodendrocytes and microglia to oxidative damage, and this is of great importance for potential therapeutic strategies [28,29].

Although it is uncertain that changes in peripheral TAS levels reflect TAS variations in the central nervous system, the evidence obtained in our study supports the use of peripheral TAS markers in patients with first-episode psychosis, based on the special sensitivity of the brain to oxidative damage [30]. In addition, recent studies have revealed that plasma markers correlate to cerebrospinal fluid [31]and postmortem brain tissue [32] markers, which is suggestive of a parallel and synchronized activity between the peripheral and central systems [33].

Our study has several limitations and strengths. A limitation is related to the follow-up of patients. This being a two-year longitudinal study of children and adolescents, follow-up of all patients was difficult. On the other hand, the strengths of the study include that the sample was highly homogeneous in terms of age, duration of the illness, and pharmacological treatment, and that a long-term follow-up was conducted. Further longitudinal studies should be performed in the future to determine the antioxidant status of patients with FEP and its potential relationship with the transcription factor Nrf2; this would allow to investigate the pathophysiology of this condition and identify new pharmacological targets.

## Conclusions

In brief, our data indicates that, in the early stages of the illness, FEP patients with a poorer clinical status have a lower antioxidant capacity but in the long term, this correlation is reversed and their antioxidant defence capacity seems to improve as a compensatory response mechanism of the body. This finding should be seriously considered, as it suggests that the antioxidant treatments currently under study should be only applied during the early stages of the illness or, at least, their long-term use is more questionable.

## Supporting information

S1 Table Potential confounding factors tested in in the three TAS measurements. NA = not applicable.
(DOCX)

S1 Fig Relationship between total antioxidant status (TAS) and Young Mania Rating Scale (YMRS) scores over a two year follow-up. (TIF)

S2 Fig Relationship between total antioxidant status (TAS) and the positive syndrome scale (PANSS Pos) scores over a two year follow-up. (TIF)

## References

[1] Bitanihirwe BKY, Woo T-UW. Oxidative stress in schizophrenia: an integrated approach. Neurosci Biobehav Rev. 2011;35: 878–893. doi: 10.1016/j.neubiorev.2010.10.008 20974172 10.1016/j.neubiorev.2010.10.008 PMC3021756

[2] Do KQ, Trabesinger AH, Kirsten-Krüger M, Lauer CJ, Dydak U, Hell D, et al Schizophrenia: glutathione deficit in cerebrospinal fluid and prefrontal cortex in vivo. Eur J Neurosci. 2000;12: 3721–3728. 11029642 10.1046/j.1460-9568.2000.00229.x

[3] Fendri C, Mechri A, Khiari G, Othman A, Kerkeni A, Gaha L. [Oxidative stress involvement in schizophrenia pathophysiology: a review]. L’Encéphale. 2006;32: 244–252. 16910626 10.1016/s0013-7006(06)76151-6

[4] Miyaoka T, Yasukawa R, Yasuda H, Shimizu M, Mizuno S, Sukegawa T, et al Urinary excretion of biopyrrins, oxidative metabolites of bilirubin, increases in patients with psychiatric disorders. Eur Neuropsychopharmacol. 2005;15: 249–252. doi: 10.1016/j.euroneuro.2004.11.002 15820412 10.1016/j.euroneuro.2004.11.002

[5] Sawa A, Sedlak TW. Oxidative stress and inflammation in schizophrenia. Schizophr Res. 2016;176: 1–2. doi: 10.1016/j.schres.2016.06.014 27395767 10.1016/j.schres.2016.06.014

[6] Kolosova NG, Shcheglova TV, Sergeeva SV, Loskutova LV. Long-term antioxidant supplementation attenuates oxidative stress markers and cognitive deficits in senescent-accelerated OXYS rats. Neurobiol Aging. 2006;27: 1289–1297. doi: 10.1016/j.neurobiolaging.2005.07.022 16246464 10.1016/j.neurobiolaging.2005.07.022

[7] Gama CS, Salvador M, Andreazza AC, Kapczinski F, Silva Belmonte-de-Abreu P. Elevated serum superoxide dismutase and thiobarbituric acid reactive substances in schizophrenia: a study of patients treated with haloperidol or clozapine. Prog Neuropsychopharmacol Biol Psychiatry. 2006;30: 512–515. doi: 10.1016/j.pnpbp.2005.11.009 16426720 10.1016/j.pnpbp.2005.11.009

[8] Gama CS, Andreazza AC, Kunz M, Berk M, Belmonte-de-Abreu PS, Kapczinski F. Serum levels of brain-derived neurotrophic factor in patients with schizophrenia and bipolar disorder. Neurosci Lett. 2007;420: 45–48. doi: 10.1016/j.neulet.2007.04.001 17442489 10.1016/j.neulet.2007.04.001

[9] Khandaker GM, Cousins L, Deakin J, Lennox BR, Yolken R, Jones PB. Inflammation and immunity in schizophrenia: implications for pathophysiology and treatment. Lancet Psychiatry. 2015;2: 258–270. doi: 10.1016/S2215-0366(14)00122-9 26359903 10.1016/S2215-0366(14)00122-9 PMC4595998

[10] Micó JA, Rojas-Corrales MO, Gibert-Rahola J, Parellada M, Moreno D, Fraguas D, et al Reduced antioxidant defense in early onset first-episode psychosis: a case-control study. BMC Psychiatry. 2011;11: 26 doi: 10.1186/1471-244X-11-26 21320302 10.1186/1471-244X-11-26 PMC3045298

[11] Raffa M, Atig F, Mhalla A, Kerkeni A, Mechri A. Decreased glutathione levels and impaired antioxidant enzyme activities in drug-naive first-episode schizophrenic patients. BMC Psychiatry. 2011;11: 124 doi: 10.1186/1471-244X-11-124 21810251 10.1186/1471-244X-11-124 PMC3161936

[12] Zhang XY, Chen DC, Xiu MH, Tang W, Zhang F, Liu L, et al Plasma total antioxidant status and cognitive impairments in schizophrenia. Schizophr Res. 2012;139: 66–72. doi: 10.1016/j.schres.2012.04.009 22555016 10.1016/j.schres.2012.04.009

[13] Tunçel ÖK, Sarısoy G, Bilgici B, Pazvantoglu O, Çetin E, Ünverdi E, et al Oxidative stress in bipolar and schizophrenia patients. Psychiatry Res. 2015;228: 688–694. doi: 10.1016/j.psychres.2015.04.046 26117246 10.1016/j.psychres.2015.04.046

[14] Martínez-Cengotitabengoa M, Micó JA, Arango C, Castro-Fornieles J, Graell M, Payá B, et al Basal low antioxidant capacity correlates with cognitive deficits in early onset psychosis. A 2-year follow-up study. Schizophr Res. 2014;156: 23–29. doi: 10.1016/j.schres.2014.03.025 24768133 10.1016/j.schres.2014.03.025

[15] Sánchez-Rodríguez MA, Santiago E, Arronte-Rosales A, Vargas-Guadarrama LA, Mendoza-Núñez VM. Relationship between oxidative stress and cognitive impairment in the elderly of rural vs. urban communities. Life Sci. 2006;78: 1682–1687. doi: 10.1016/j.lfs.2005.08.007 16246376 10.1016/j.lfs.2005.08.007

[16] Yao JK, Reddy R, McElhinny LG, van Kammen DP. Reduced status of plasma total antioxidant capacity in schizophrenia. Schizophr Res. 1998;32: 1–8. doi: 10.1016/S0920-9964(98)00030-9 9690328 10.1016/s0920-9964(98)00030-9

[17] Castro-Fornieles J, Parellada M, Gonzalez-Pinto A, Moreno D, Graell M, Baeza I, et al The child and adolescent first-episode psychosis study (CAFEPS): design and baseline results. Schizophr Res. 2007;91: 226–237. doi: 10.1016/j.schres.2006.12.004 17267179 10.1016/j.schres.2006.12.004

[18] Peralta Martín V, Cuesta Zorita MJ. [Validation of positive and negative symptom scale (PANSS) in a sample of Spanish schizophrenic patients]. Actas Luso-Esp Neurol Psiquiatr Cienc Afines. 1994;22: 171–177. 7810373

[19] Hamilton M. Development of a rating scale for primary depressive illness. Br J Soc Clin Psychol. 1967;6: 278–296. 6080235 10.1111/j.2044-8260.1967.tb00530.x

[20] Young RC, Biggs JT, Ziegler VE, Meyer DA. A rating scale for mania: reliability, validity and sensitivity. Br J Psychiatry J Ment Sci. 1978;133: 429–435. 10.1192/bjp.133.5.429 728692

[21] Shaffer D, Gould MS, Brasic J, Ambrosini P, Fisher P, Bird H, et al A children’s global assessment scale (CGAS). Arch Gen Psychiatry. 1983;40: 1228–1231. 6639293 10.1001/archpsyc.1983.01790100074010

[22] Re R, Pellegrini N, Proteggente A, Pannala A, Yang M, Rice-Evans C. Antioxidant activity applying an improved ABTS radical cation decolorization assay. Free Radic Biol Med. 1999;26: 1231–1237. 10381194 10.1016/s0891-5849(98)00315-3

[23] Mauerhofer C, Philippova M, Oskolkova OV, Bochkov VN. Hormetic and anti-inflammatory properties of oxidized phospholipids. Mol Aspects Med. 2016; doi: 10.1016/j.mam.2016.02.003 26948981 10.1016/j.mam.2016.02.003

[24] Martinez-Cengotitabengoa M, MacDowell KS, Alberich S, Diaz FJ, Garcia-Bueno B, Rodriguez-Jimenez R, et al BDNF and NGF Signalling in Early Phases of Psychosis: Relationship With Inflammation and Response to Antipsychotics After 1 Year. Schizophr Bull. 2016;42: 142–151. doi: 10.1093/schbul/sbv078 26130821 10.1093/schbul/sbv078 PMC4681544

[25] Mendoza-Núñez VM, Sánchez-Rodríguez MA, Retana-Ugalde R, Vargas-Guadarrama LA, Altamirano-Lozano MA. Total antioxidant levels, gender, and age as risk factors for DNA damage in lymphocytes of the elderly. Mech Ageing Dev. 2001;122: 835–847. 11337012 10.1016/s0047-6374(01)00240-8

[26] Bartolini D, Galli F. The functional interactome of GSTP: A regulatory biomolecular network at the interface with the Nrf2 adaption response to oxidative stress. J Chromatogr B Analyt Technol Biomed Life Sci. 2016; doi: 10.1016/j.jchromb.2016.02.002 26922696 10.1016/j.jchromb.2016.02.002

[27] Jiang S, Deng C, Lv J, Fan C, Hu W, Di S, et al Nrf2 Weaves an Elaborate Network of Neuroprotection Against Stroke. Mol Neurobiol. 2016; doi: 10.1007/s12035-016-9707-7 26846360 10.1007/s12035-016-9707-7

[28] Zhao X, Aronowski J. Nrf2 to pre-condition the brain against injury caused by products of hemolysis after ICH. Transl Stroke Res. 2013;4: 71–75. doi: 10.1007/s12975-012-0245-y 23378859 10.1007/s12975-012-0245-y PMC3558942

[29] Summergrad P. Investing in global mental health: the time for action is now. Lancet Psychiatry. 2016;3: 390–391. doi: 10.1016/S2215-0366(16)30031-1 27083118 10.1016/S2215-0366(16)30031-1

[30] Ng F, Berk M, Dean O, Bush AI. Oxidative stress in psychiatric disorders: evidence base and therapeutic implications. Int J Neuropsychopharmacol. 2008;11: 851–876. doi: 10.1017/S1461145707008401 18205981 10.1017/S1461145707008401

[31] Coughlin JM, Wang Y, Ambinder EB, Ward RE, Minn I, Vranesic M, et al In vivo markers of inflammatory response in recent-onset schizophrenia: a combined study using [(11)C]DPA-713 PET and analysis of CSF and plasma. Transl Psychiatry. 2016;6: e777 doi: 10.1038/tp.2016.40 27070405 10.1038/tp.2016.40 PMC4872398

[32] Harris LW, Pietsch S, Cheng TMK, Schwarz E, Guest PC, Bahn S. Comparison of peripheral and central schizophrenia biomarker profiles. PloS One. 2012;7: e46368 doi: 10.1371/journal.pone.0046368 23118852 10.1371/journal.pone.0046368 PMC3484150

[33] Fernandes BS, Steiner J, Bernstein H-G, Dodd S, Pasco JA, Dean OM, et al C-reactive protein is increased in schizophrenia but is not altered by antipsychotics: meta-analysis and implications. Mol Psychiatry. 2016;21: 554–564. doi: 10.1038/mp.2015.87 26169974 10.1038/mp.2015.87

