# Supplementary material for: The complex association between the antioxidant defense system and clinical status in early psychosis
Source: PLoS One. 2018 Apr 26;13(4):e0194685. doi: 10.1371/journal.pone.0194685 (PMC5919675; doi:10.1371/journal.pone.0194685)
Supplement: S1 Table — NA = not applicable. (DOCX) [file pone.0194685.s001.docx]

| **Sociodemographic characteristics** | **BASELINE TAS** | **1 YEAR TAS** | **2 YEAR TAS** |
| --- | --- | --- | --- |
|  |  |  |  |
| Age | r= -0.19; p= 0.11 | r= 0.02; p= 0.92 | r= -0.23; p =0.24 |
| Sex | t= -1.18; p= 0.24 | t= -1.63; p= 0.11 | t= -1.59; p =0.12 |
| Ethnic group | F= 0.64; p= 0.53 | F= 0.19; p= 0.82 | F= 0.28; p =0.59 |
| Socioeconomic status | F= 1.42; p= 0.24 | F= 1.95; p= 0.12 | F= 1.12; p =0.37 |
| Type of living arrangement | F= 1.20; p= 0.31 | F= 0.67; p= 0.52 | F= 1.76; p =0.20 |
|  |  |  |  |
| Antipsychotic dose |  |  |  |
| *Baseline* | r= -0.14; p =0.27 | NA | NA |
| *1 year* | NA | r= 0.09; p= 0.61 | NA |
| *2 year* | NA | NA | r= 0.033; p= 0.90 |
|  |  |  |  |
| Tobacco |  |  |  |
| *baseline* | t= -0.84; p= 0.40 | t= -0.31; p= 0.76 | t= -0.92; p= 0.37 |
| *1 year* | NA | t= -0.50; p= 0.62 | t= -0.09; p= 0.93 |
| *2 year* | NA | NA | t= 0.39; p= 0.69 |
|  |  |  |  |
| Cannabis |  |  |  |
| *Baseline* | t= -1.62; p= 0.11 | t= -0.11; p= 0.91 | t= 0.08; p= 0.94 |
| *1 year* | NA | t= -0.28; p= 0.78 | t= -0.85; p= 0.40 |
| *2 year* | NA | NA | t= 0.01; p= 0.99 |
|  |  |  |  |
| Alcohol |  |  |  |
| *Baseline* | t= 0.64; p= 0.52 | t= -0.49; p= 0.62 | t= -0.68; p= 0.49 |
| *1 year* | NA | t= -0.39; p= 0.69 | t= 0.33; p= 0.74 |
| *2 year* | NA | NA | t= 0.30; p= 0.77 |

S1 Table. Potential confounding factors tested in the three TAS measurements

*NA= not applicable*
