# Supplementary figures and images for: The complex association between the antioxidant defense system and clinical status in early psychosis
Source: PLoS One. 2018 Apr 26;13(4):e0194685. doi: 10.1371/journal.pone.0194685 (PMC5919675; doi:10.1371/journal.pone.0194685)

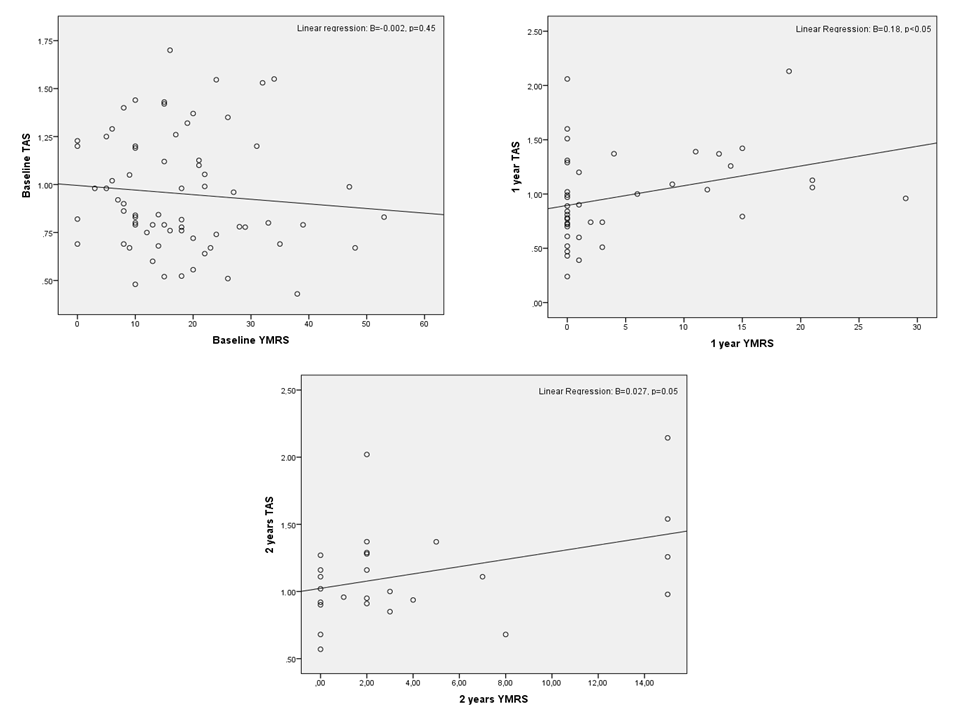

Supplement: S1 Fig — (TIF) [file pone.0194685.s002.tif]

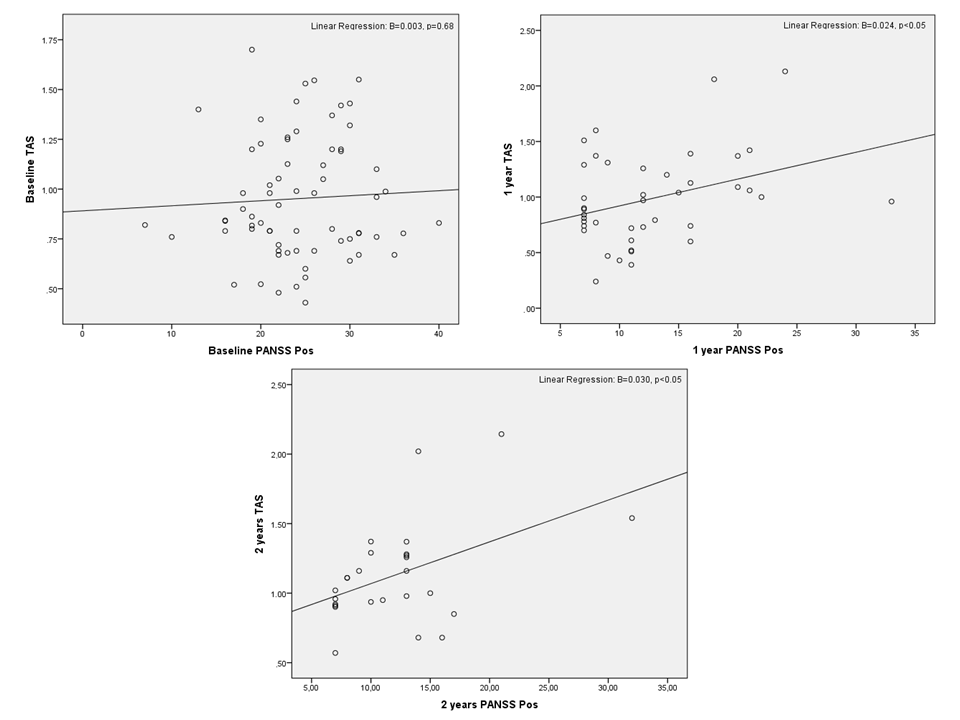

Supplement: S2 Fig — (TIF) [file pone.0194685.s003.tif]
